# Semantic segmentation of microbial alterations based on SegFormer

**DOI:** 10.3389/fpls.2024.1352935

**Published:** 2024-06-13

**Authors:** Wael M. Elmessery, Danil V. Maklakov, Tamer M. El-Messery, Denis A. Baranenko, Joaquín Gutiérrez, Mahmoud Y. Shams, Tarek Abd El-Hafeez, Salah Elsayed, Sadeq K. Alhag, Farahat S. Moghanm, Maksim A. Mulyukin, Yuliya Yu. Petrova, Abdallah E. Elwakeel

**Affiliations:** ^1^ Agricultural Engineering Department, Faculty of Agriculture, Kafrelsheikh University, Kafr El-Sheikh, Egypt; ^2^ Engineering Group, Centro de Investigaciones Biológicas del Noroeste, La Paz, Baja California Sur, Mexico; ^3^ International Research Centre “Biotechnologies of the Third Millennium”, Faculty of Biotechnologies (BioTech), ITMO University, St. Petersburg, Russia; ^4^ Department of Machine Learning and Information Retrieval, Faculty of Artificial Intelligence, Kafrelsheikh University, Kafr El-Sheikh, Egypt; ^5^ Department of Computer Science, Faculty of Science, Minia University, Minia, Egypt; ^6^ Computer Science Unit, Deraya University, Minia University, Minia, Egypt; ^7^ Agricultural Engineering, Evaluation of Natural Resources Department, Environmental Studies and Research Institute, University of Sadat City, Sadat City, Egypt; ^8^ Biology Department, College of Science and Arts, King Khalid University, Abha, Saudi Arabia; ^9^ Soil and Water Department, Faculty of Agriculture, Kafrelsheikh University, Kafr El-Sheikh, Egypt; ^10^ Institute of Natural and Technical Sciences, Surgut State University, Surgut, Russia; ^11^ Agricultural Engineering Department, Faculty of Agriculture and Natural Resources, Aswan University, Aswan, Egypt

**Keywords:** computer vision, mix transformer encoders, disease detection, smart agriculture, food safety

## Abstract

**Introduction:**

Precise semantic segmentation of microbial alterations is paramount for their evaluation and treatment. This study focuses on harnessing the SegFormer segmentation model for precise semantic segmentation of strawberry diseases, aiming to improve disease detection accuracy under natural acquisition conditions.

**Methods:**

Three distinct Mix Transformer encoders - MiT-B0, MiT-B3, and MiT-B5 - were thoroughly analyzed to enhance disease detection, targeting diseases such as Angular leaf spot, Anthracnose rot, Blossom blight, Gray mold, Leaf spot, Powdery mildew on fruit, and Powdery mildew on leaves. The dataset consisted of 2,450 raw images, expanded to 4,574 augmented images. The Segment Anything Model integrated into the Roboflow annotation tool facilitated efficient annotation and dataset preparation.

**Results:**

The results reveal that MiT-B0 demonstrates balanced but slightly overfitting behavior, MiT-B3 adapts rapidly with consistent training and validation performance, and MiT-B5 offers efficient learning with occasional fluctuations, providing robust performance. MiT-B3 and MiT-B5 consistently outperformed MiT-B0 across disease types, with MiT-B5 achieving the most precise segmentation in general.

**Discussion:**

The findings provide key insights for researchers to select the most suitable encoder for disease detection applications, propelling the field forward for further investigation. The success in strawberry disease analysis suggests potential for extending this approach to other crops and diseases, paving the way for future research and interdisciplinary collaboration.

## 1 Introduction

As artificial intelligence continues to find applications in diverse domains, the field of agricultural science is no exception. Computer vision methodologies have been introduced to various tasks related to plant image analysis. These tasks encompass plant classification, as demonstrated in the works of Barre et al. (2017) and Wäldchen and Mäder (2018), as well as the detection of plant diseases and pests, as evidenced by Shruthi et al. (2019) and Chouhan et al. (2020).

The detection of plant diseases and pests has garnered substantial interest, mainly centering around deep-learning-driven computer vision techniques. Distinct from traditional computer vision models that rely on human-crafted image features, these modern approaches display enhanced robustness to environmental disparities, attributable to extensive training on expansive datasets. The Egyptian agricultural economy has witnessed a surge in prominence pertaining to strawberry farming, attributed largely to the nation’s auspicious climate and fertile lands located in regions like Wadi El Natroun, El Beheira, and Fayoum. Ideal for strawberry cultivation, these territories accommodate bountiful harvests annually from November to April. Spearheading strawberry production in the Middle East and North African region, Egypt recorded a yield of approximately 597.03 thousand tons in 2020. Export trends indicate a steady flow of strawberry shipments, predominantly directed towards European markets, culminating in a figure of 24.72 thousand tons in 2022 (TRIDGE, 2023).

While the strawberry industry in Egypt has experienced growth, it faces certain challenges, such as the need for improved pest and disease management practices. Detecting plant diseases at their initial stages can significantly reduce the need for potentially harmful chemicals and minimize labor expenses associated with managing afflicted plants. Even experienced farmers can face challenges in identifying diseases in large greenhouse settings before they propagate. Hence, an automated disease detection system will serve as a valuable complement to farmers’ expertise and effort. Timely detection and accurate identification of pests are crucial not only for preventing crop damage, but also for avoiding the incorrect and excessive application of pesticide sprays (Dong et al., 2021). From the analysis of various datasets related to strawberry diseases, we have identified the presence of seven distinct diseases: Leaf spot (*Mycosphaerella fragariae*), Angular leaf spot (*Xanthomonas fragariae*), Anthracnose rot (*Colletotrichum acutatum*), Blossom blight (*Monilinia fructicola*), Gray mold (*Botrytis cinerea)*, Powdery mildew on fruit (*Podosphaera aphanis*), and Powdery mildew on leaves (*Podosphaera macularis*). Efficient and accurate segmentation of leaf disease represents a significant area of research. To tackle this challenge, a wide range of computer vision segmentation methods have been employed, leveraging image attributes like hue, texture, form, and spatial information (Pugoy and Mariano, 2011; Revathi and Hemalatha, 2012; Wang et al., 2018; Deenan et al., 2020; Zhao et al., 2020). However, these conventional techniques come with inherent limitations and typically require a significant amount of time. The emergence of deep learning models marks a transformative era for segmenting images. Li et al. (2023) introduced a network grounded in copy-paste techniques and SegFormer, showcasing its prowess in precise segmentation of disease regions and evaluation of their severity, marked by mean intersection over union of 85.38%. Wu et al. (2023) further refined the landscape by enhancing DETR, leading to the efficient segmentation of tomato leaf disease spots and achieving an impressive accuracy of 96.40%. Zhao et al. (2022) proposed a multiple disease detection method for greenhouse-cultivated strawberry based on multiscale feature fusion Faster R-CNN.

In a comprehensive investigation conducted by (Minaee et al., 2020) an extensive evaluation of segmentation approaches based on deep learning presented in 2019 was carried out. Notably, Convolutional Neural Networks (CNNs) have been extensively utilized in tasks related to the segmentation of agricultural diseases. They have proven instrumental in enhancing the precision of disease spot identification and significantly expanding the range of potential applications (Jiang et al., 2020; Craze et al., 2022; Yao et al., 2022; Yong et al., 2023).

Transformers have shown superior performance compared to convolutional neural networks, achieving state-of-the-art results with fewer parameters and higher computational efficiency (Fan and Liu, 2023). Transformers, particularly self-attention modules, provide efficient object detection models and improve detection accuracy in deep foggy conditions (Shit et al., 2023). They also offer consistent, albeit modest, performance improvements when added to state-of-the-art segmentation models for overhead imagery (Luzi et al., 2023). However, transformers have some limitations. They are difficult to train and have lower performance on small datasets compared to convolutional neural networks (Chen and Feng, 2023). Fully transformer-based models may achieve relatively poor performance, while hybrid models that combine convolutional and transformer-based structures show better results.

Transformer-based architectures can be adapted to handle other visual tasks, such as object detection and segmentation, by leveraging their self-attention mechanism and hierarchical feature representation capabilities. These architectures have shown remarkable advancements in visual segmentation tasks, surpassing previous convolutional or recurrent approaches (Gao et al., 2023).

In the realm of semantic segmentation for agricultural diseases, a series of transformative visual networks based on Transformers has unfolded, showcasing notable advancements. The journey begins with the inception of models like Detection Transformer (DETR) (Carion et al., 2020), Vision Transformer (ViT) (Dosovitskiy et al., 2020), Swin Transformer (SwinT) (Liu et al., 2021), Semantic Transformation model (SETR) (Zheng et al., 2021), and SegFormer (Xie et al., 2021). Building on this foundation (Wang et al., 2022), elevated the SwinT network, employing it for identifying real plant leaf diseases, (Wu et al., 2022) further refined the landscape by enhancing DETR, leading to the efficient segmentation of tomato leaf disease spots and achieving an impressive accuracy of 96.40%. (Reedha et al., 2022) took a visionary leap by applying vision transformer (ViT) for categorizing weeds and crop images obtained from agricultural drones, outperforming traditional CNNs with an outstanding F1 score of 99.28%. In a pursuit of lightweight yet effective solutions, (Li et al., 2022) introduced a network grounded in copy–paste techniques and SegFormer, showcasing its prowess in precise segmentation of disease regions and evaluation of their severity, marked by mean intersection over union of 85.38%. The narrative unfolds further with (Zhang et al., 2023), who suggested a specialized segmentation framework known as the Cross-Resolution Transformer, tailored for identifying the leaf disease of the grape in natural environments. Through these transformative steps, SegFormer emerges as a straightforward, effective, and resilient framework for semantic segmentation unifying Transformers with nimble multi-layer perceptron decoders, thereby contributing significantly to the evolving landscape of agricultural disease segmentation.

### 1.1 Problem statement

Precise detection and segmentation of strawberry diseases are crucial for effective management and treatment. Traditional computer vision methods often fall short in accurately identifying diseases, particularly under natural acquisition conditions. Deep learning models, especially transformer-based architectures like SegFormer, offer promising solutions. However, selecting an appropriate mix transformer encoder for optimal performance remains a challenge. Moreover, the existing studies often lack in-depth analysis and comparison of different encoder variants in the context of disease detection accuracy. Therefore, this study aims to address these gaps by evaluating and enhancing the SegFormer segmentation model using three distinct Mix Transformer encoders (MiT-B0, MiT-B3, and MiT-B5) for precise identification and localization of various strawberry diseases.

### 1.2 Contributions

This study explores the potential of SegFormer, a powerful segmentation model, for accurately detecting and distinguishing seven strawberry diseases. Three Mix Transformer encoders within SegFormer were investigated and their performance, adaptability, and impact on disease detection were analyzed. The main contributions can be summarized as follows:

- **Hybrid model design:** A novel hybrid model leverages the strengths of both Mix Transformer encoders and SegFormer architecture for effective disease segmentation while mitigating overfitting and generalization issues.
- **Extensive dataset:** Experiments are conducted on a diverse dataset of 4,574 augmented images, ensuring balanced class representation and enabling robust performance assessment under various disease scenarios.
- **Quantitative and qualitative results:** Using metrics like mIoU and MPA, superior performance compared to the existing methods is demonstrated. Visual examples further confirm the model’s robustness and practical value.
- **State-of-the-art performance:** This approach achieves outstanding accuracy, efficiency, and reduced model complexity compared to the established models, making SegFormer a strong contender for real-world applications in strawberry disease detection.
- **Insights and future directions:** Valuable insights into the relationship between encoders and SegFormer performance are provided, guiding researchers in model fine-tuning and tailored strategies for diverse agricultural challenges.
- **Wider applicability:** The success in strawberry disease analysis suggests potential for extending this approach to other crops and diseases, paving the way for future research and interdisciplinary collaboration.

The remainder of the paper is structured as follows: Section 2 reviews previous research to provide context and familiarize readers with the current state of knowledge in the field. Section 3 delves into the materials, methods, and specifics of the proposed model, laying the groundwork for understanding the subsequent experiments. In Section 4, experimental results are presented to demonstrate the proposed model’s performance under various conditions. Section 5 discusses limitations encountered during the research process, promoting transparency and encouraging critical examination. Finally, Section 6 consolidates conclusions drawn from the experimental results and suggests potential avenues for future research.

### 1.3 Related work

The research evaluating the severity of plant diseases using Convolutional Neural Networks (CNNs) primarily focused on two main approaches. The first category involves techniques centered around image segmentation, while the second focuses on enhancing CNNs, predominantly by incorporating the Attention Mechanism (Naga Srinivasu et al., 2020).

Segmentation-based methods typically utilize popular segmentation networks like DeepLabV3+, U-Net, PSPNet, and Mask R-CNN. For instance, Wang et al. (2022) refined the SwinT network for data augmentation and identifying actual cucumber leaf diseases. Meanwhile, Wu et al. (2022) obtained a remarkable disease classification accuracy of 96.40% for *tomat* eaf diseases by implementing various improvements to DETR. Additionally, Reedha et al. (2022) leveraged ViT to classify weed and crop images acquired via Unmanned Aerial Vehicles, resulting in an outstanding F1 score of 99.28%. Lastly, Li et al. (2022) put forth a lightweight network grounded in copy–paste and SegFormer for precise disease-region segmentation and severity assessment, yielding a MIoU of 85.38%.

Aside from segmentation-focused methods, researchers explored alternative ways to improve CNNs, mainly concentrating on introducing the Attention Mechanism. Zhang et al. (2023) utilized a three-stage methodology to classify “Huangguan” pears. Initially, Mask R-CNN facilitated the segmentation of “Huangguan” pears from intricate backdrops; subsequently, DeepLabV3+, U-Net, and PSPNet served to segment “Huangguan” pear spots, calculating the proportion of spot area relative to the total number of pixels. This ratio was classified into three distinct grades. During the final phase, ResNet-50, VGG-16, and MobileNetV3 contributed to determining the pear’s condition level.

Liu et al. (2021) applied a staged segmentation concept. First, they separated apple leaves from complicated environments using a deep learning algorithm before detecting the affected regions on the isolated leaves. Subsequently, they gauged the severity of illnesses by computing the ratio of damaged tissue to the entire leaf area.

Moreover, the Attention Mechanism gained prominence in recent studies. Yin et al. (2022) modified the DCNN through integration of multi-scale and Attention Mechanisms, ultimately realizing maize small leaf spot classification. Separately, Liu et al. (2021) combined multi-scale convolution kernels and Coordinate Attention Mechanism in SqueezeNext to estimate illness severity, leading to a 3.02% improvement over the initial SqueezeNext model.

## 2 Materials and methods

### 2.1 Experimental environment

In this study, publicly accessible datasets were utilized, specifically the Kaggle Dataset (Afzaal et al., 2021), to create a customized dataset tailored to the training and evaluation requirements of this study. The input image size was standardized to 128x128 pixels. However, it is important to note that the original images in the dataset had varying resolutions. Initially, the Kaggle Dataset comprised 1972 images encompassing seven distinct strawberry diseases. By employing an augmentation process, the overall dataset size was substantially expanded, resulting in a total of 4574 images available in two resolutions: 512 X 512 pixels and 640 X 640 pixels. **Table 1** demonstrates a detailed breakdown of how these images were distributed across various disease categories, which provides a comprehensive overview of the dataset’s composition.

**Table 1:** Statistics of the Raw and Augmented Datasets.

Disease No.	Disease name	Raw Images count	Augmented images count
1	Angular leaf spot	245	569
2	Anthracnose fruit rot	52–156	118–354
3	Blossom blight	119–357	273–819
4	Gray mold	254	606
5	Leaf spot	369	919
6	Powdery mildew fruit	79–273	185–555
7	Powdery mildew leaf	318	752

### 2.2 Dataset annotation and preparation

In this study, the innovative Segment Anything Model (SAM) integrated into the Roboflow annotation tool (Roboflow, 2023) was utilized to expedite the annotation and preparation of a strawberry disease dataset. This integration allowed for swift annotation of complex strawberry disease instances using a smart polygon tool in the Roboflow editor. SAM demonstrated proficiency in handling intricate object boundaries found in various disease manifestations, enabling the efficient creation of accurate segmentation masks. This approach not only saved considerable time, but also ensured the precision and quality of the annotations. The integration of SAM into the Roboflow annotation tool proved to be a valuable asset, simplifying data preparation and enhancing the accuracy of the semantic segmentation task in this research.

### 2.3 Dataset Augmentation and preprocessing

**Figure 1:**
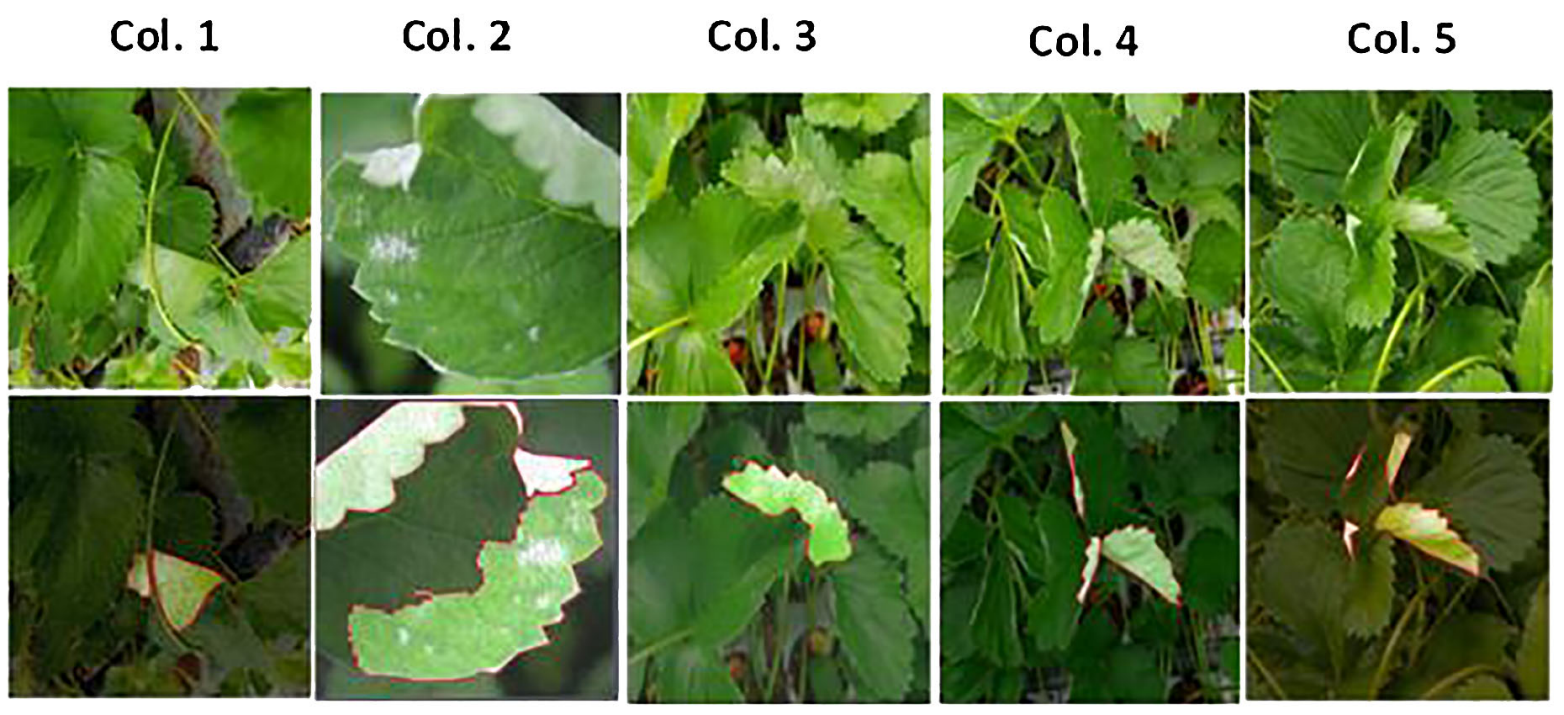
Representative example of data augmentation scenarios on powdery mildew on leaves. The top row shows the original images, while the bottom row illustrates the images after resizing and data enrichment procedures. The first column displays images after adjustments, including a decrease in hue by -8°, saturation by -1%, brightness by -12%, along with a 1px blur and 8% noise. The second column presents images after horizontal flipping, a 2° hue increase, 5% saturation increase, 23% brightness increase, a 1.75px blur, and 3.25% noise. In the third column, images are shown following an increase in hue by 19°, a 2% saturation boost, 23% brightness enhancement, along with 0.5px blur and 0.75% noise. The fourth column depicts images after a 19° hue increase, and the fifth column shows images with a -18° reduction in hue.

A comprehensive set of augmentation techniques was employed to enhance the quality and diversity of the strawberry diseases’ dataset. Data augmentation was performed in all sets of training, validation and test. The augmentation processes included horizontal flips, which help the model adapt to different orientations. Additionally, hue adjustments within the range of -21 to +21°, saturation variations from -5% to +5%, and brightness changes spanning from -25% to +25% were applied. These modifications contribute to the dataset robustness by simulating different lighting conditions and color variations. To introduce realistic imperfections, a blur with a maximum radius of 2.5 pixels and introduced noise, affecting up to 8% of the pixels, was incorporated. **Figure 1** illustrates a representative example of applying various augmentation scenarios to powdery mildew leaf images. These augmentation strategies are presented in **Table 2** and play a crucial role in improving the dataset variability and aiding the proposed SegFormer-based semantic segmentation model in effectively recognizing and classifying strawberry diseases.

**Table 2:** Augmentation methods and their respective settings.

Method	Settings
Flip	Horizontal
Hue	Between -21° and +21°
Saturation	Between -5 and +5%
Brightness	Between -25 and +25%
Blur	Up to 2.5px
Noise	Up to 8% of pixels

The process of dividing a dataset into training, validation, and test subsets is a fundamental step in deep learning model development, ensuring the model’s generalizability and performance evaluation. In this study, a diverse dataset containing various plant diseases was analyzed. To achieve a balanced and representative split, the size of each class was considered. With 569 images of Angular Leaf Spot, 354 images of Anthracnose Fruit Rot, 819 images of Blossom Blight, 606 images of Gray Mold, 919 images of Leaf Spot, 555 images of Powdery Mildew Fruit, and 752 images of Powdery Mildew Leaf, the data were appropriately distributed. Typically, a common practice is to allocate a significant portion of the dataset to training, around 80–90%, to allow the model to learn from a substantial amount of data. The validation set, which is usually 5–10% of the data, is employed during model development to fine-tune hyperparameters and monitor training progress. The remaining portion, the test set, serves as an unseen dataset to evaluate the model performance objectively, as illustrated in **Table 3**.

**Table 3:** Dataset distribution.

Disease type	Total samples	Training size	Validation size	Test size
Angular leaf spot	569	514	37	18
Anthracnose fruit rot	354	310	30	14
Blossom blight	273	225	26	18
Gray mold	606	534	58	18
Leaf spot	919	765	74	40
Powdery mildew fruit	555	470	50	33
Powdery mildew leaf	752	651	64	37

### 2.4 Efficient Segmentation model training with PyTorch Lightning Framework

In this study, PyTorch Lightning was employed as a powerful deep learning framework to train a semantic segmentation model on a strawberry diseases dataset. PyTorch Lightning provided a streamlined and highly efficient platform for the training process. It abstracted the underlying complexities of training, concentrating on model architecture and experimentation. The use of Lightning structured training loops and integrated callbacks, such as early stopping and model checkpointing, enhanced productivity, while its built-in support for distributed training and reproducibility contributed to the robustness of this research. The resulting model, based on the Segformer architecture, demonstrated impressive performance in semantic segmentation, making PyTorch Lightning an invaluable component of the methodology of the study.

### 2.5 Early Stopping and model checkpointing

Two crucial techniques were employed in this study for enhancing the training of deep learning models: Early Stopping and Model Checkpointing. The Early Stopping callback is an invaluable addition to the training regimen. It continuously monitors the validation loss as the model learns, and its role is to identify when the progress plateaus. This is defined by such parameters as ‘min_delta,’ which specifies the minimum change in validation loss to be considered as a meaningful improvement. If no substantial improvement is observed for a predefined number of consecutive epochs, set at 10 in the present study, Early Stopping steps in and terminates the training process, preventing unnecessary overfitting and saving valuable computational resources.

On the other hand, ModelCheckpoint plays a pivotal role in preserving the best version of the proposed model. By specifying ‘save_top_k=1’ and monitoring the ‘val_loss,’ it ensures that only the finest Model Checkpoint, the one with the lowest validation loss, is stored. This is crucial because it safeguards the model superior performance and provides a safety net in case of unforeseen interruptions during training. The harmonious interplay of Early Stopping and Model Checkpointing allows to train the proposed deep learning model efficiently, striking a balance between performance optimization and resource management.

### 2.6 The proposed model architecture

**Figure 2:**
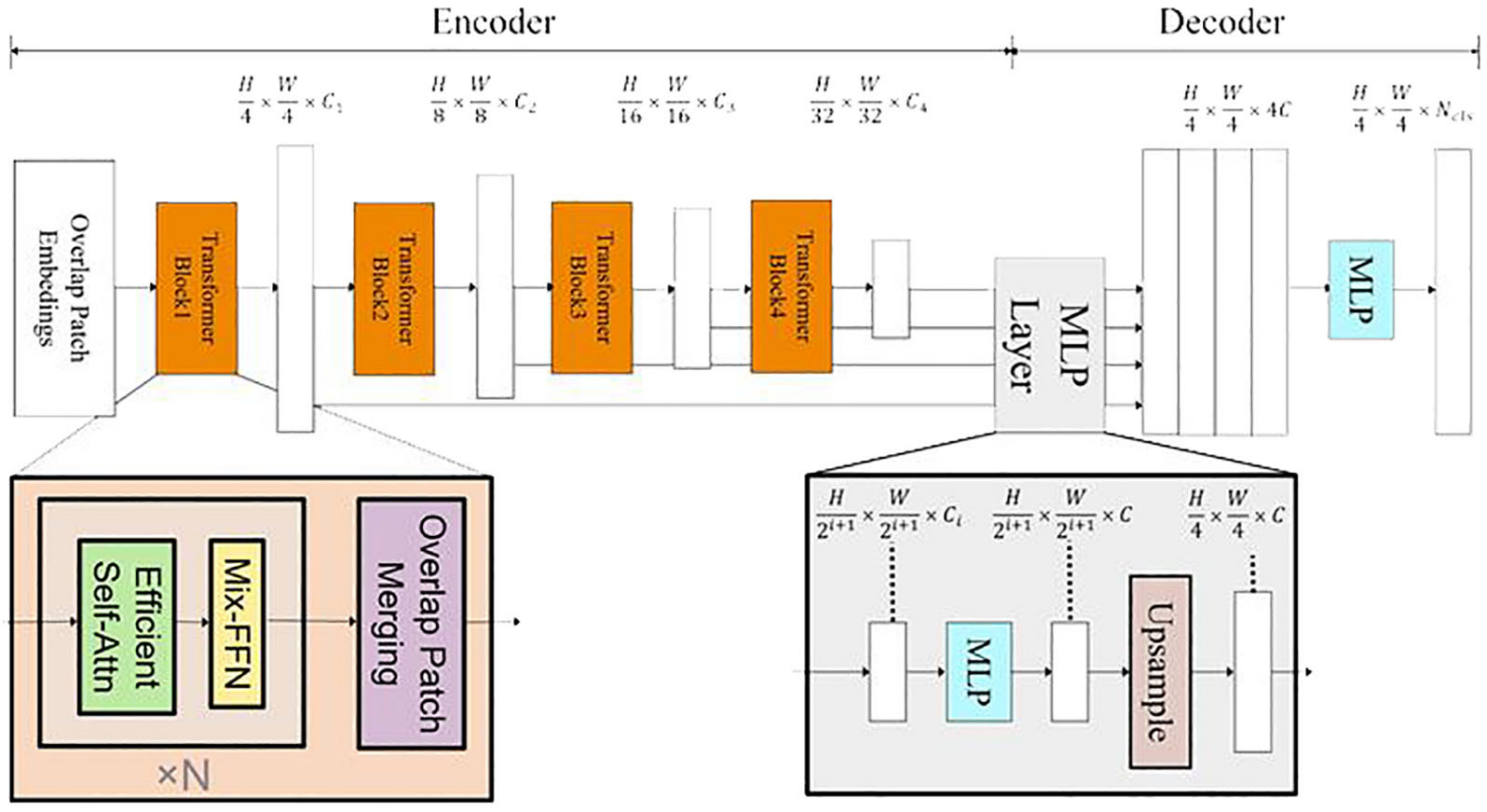
SegFormer Architecture Overview: The FFN indicates a Feed-Forward Network. H, W define the input image height and width. C defines the channel dimension in the MLP decoder and N_cls is the number of semantic classes.

In this study, the SegFormer architecture was harnessed (**Figure 2**) and fine-tuned for precise semantic segmentation and object detection. NVIDIA advanced SegFormer model, rooted in this architecture, was designed for specialized computer vision tasks. SegFormer core strength lies in its Transformer-based backbone, which excels at capturing contextual information in images. Its encoder-decoder structure and innovative Mix Feed-Forward Network (Mix-FFN) approach address positional encoding and model efficiency challenges, contributing to high-performance yet resource-efficient models.

Self-attention mechanisms, a hallmark of Transformer models, dynamically focus on relevant image regions. Fine-tuning, using a pre-trained model on the extensive ADE20K dataset, refines the model knowledge for the specific purpose of this study. The dataset diversity enhances the model proficiency in semantic segmentation and scene understanding.

For strawberry disease segmentation, MiT-B0 and MiT-B3 were tailored to handle 512x512 pixel images, while MiT-B5 was configured for 640x640 pixel images. These customizations suit the models to the unique demands of this task.

**Figure 2** presents an overview of SegFormer architectural components, which includes both encoding and decoding modules. Within the encoder, the Transformer block utilizes Overlap Patch Embeddings (OPEs) to extract feature representations and down-sample the input image. These extracted features are then fed into two critical components: the Efficient Self-Attention (ESA) and the Mix Feed-Forward Network (Mix-FFN). Here are their components and functionalities: the FFN indicates a Feed-Forward Network; H and W represent the height and width of the original image, respectively; the Transformer Block is the basic structure of the SegFormer backbone network.

To calculate the OPE, standard convolutional layers are employed. Following this, the 2D features are spatially reshaped into 1D representations and subsequently input into the ESA layer. The ESA layer plays a pivotal role in enhancing features through self-attentive computations. To address positional encoding, a 3 × 3 convolution is thoughtfully introduced between the two linear layers of the FFN. This convolutional operation effectively fuses positional information into the network.

In the encoder, Linear Normalization (LN) sequentially follows linear layers, guaranteeing normalized representation of input features. Adopting Gaussian Error Linear Units imparts non-linear properties to the model as an activation function. Crucially, the encoder deploys numerous instances of Encoding Scale-Adaptive Modules (ESAs) and Mix Feature-wise FiLM Functional Units (MixFFNs), collectively increasing the depth of the network and enabling the discovery of subtle distinctions and semantic traits. Notably, individual self-attention calculations occur at each scale inside the ESA, differing from earlier network designs executing cross-scale self-attention computations following merger via CNNs. This independent computation style improves the quality and particularity self-attention mechanisms at respective scales, enhancing pattern recognition and relationship formation.

The present research implements the assorted Mix Transformer encoders (MiT) in the model’s encoder, namely MiT-B0, MiT-B3, and MiT-B5. Classified as real-time SegFormer candidates, MiT-B0 and MiT-B3 excel in speed, while MiT-B5 adheres to the non-real-time standard favoring heightened accuracy. Outlined in **Table 4**, the principal hyperparameters of these models facilitate comparison. Experimentation entails trialing the three dissimilar SegFormer configurations to identify optimal solutions for detecting various strawberry disorders. Serving as an economical option, MiT-B0 possesses a diminished parameter count of approximately 3.4 million in the encoder and 0.4 million in the decoder. Superior performing MiT-B3 accumulates nearly 47.3 million parameters in total, representing a potent candidate amongst real-time alternatives. Further expanding upon its predecessors, MiT-B5 sports a grander configuration featuring 84.7 million parameters. The detailed comparison of the MiT encoders is shown in **Table 4**.

**Table 4:** Hyperparameters of MiT-B0, Mit-B3, and MiT-B5 architectures.

Parameters	MiT-B0	MiT-B3	MiT-B5
	Overlapping Patch embedding		
Channel number, C	[32 64 160 256]*	[64 128 320 512]	[64 128 320 512]
Patch size, K	[7 3 3 3]*	[7 3 3 3]	[7 3 3 3]
Stride, S	[4 2 2 2]*	[4 2 2 2]	[4 2 2 2]
Padding, P	[3 1 1 1]*	[3 1 1 1]	[3 1 1 1]
Transformer encoder			
Head number, N	[1 2 5 8]*	[1 2 5 8]	[1 2 5 8]
Encoder layers number, L	[2 2 2 2]*	[3 3 18 3]	[3 6 40 3]
Reduction ratio, R	[8 4 2 1]*	[8 4 2 1]	[8 4 2 1]
Expansion ratio of the feed-forward layer, E	[8 8 4 4]*	[8 8 4 4]	[8 8 4 4]
MLP decoder			
channel dimension	256	768	768
Encoder and Decoder sizes			
Encoder size, parameters	3.4	44.0	81.4
Decoder size, Parameters	0.4	3.3	3.3

*The values in the list correspond to the predefined settings for stage-1 to stage-4.

✓ The values in the list correspond to the predefined settings for stages from stage-1 to stage-4.

✓ Input tensor: typically, the SegFormer model expects input tensors with a shape of (batch_size, 3, height, width).

✓ Kernel size: convolutions within the stem layer use 3 × 3 kernels.

✓ Strides: the value is set to 1 for the majority of the layers in SegFormer.

✓ Activation function: the SegFormer model frequently employs GELU (Gaussian Error Linear Unit).

✓ The learning rate used is 0.00002.

MiT-B0 is the most compact model optimized for real-time applications, MiT-B3 is the larger model suitable for real-time tasks, and MiT-B5 is the largest model specifically designed for high-performance applications.

### 2.7 Architectural and mechanical variations between mix transformer encoders-decoders

The steps for Understanding SegFormer Variants and their operations can be summarized as follows:

1. Examine three mix transformer encoder options—MiT-B0, MiT-B3, and MiT-B5—each having different sizes, depths, and complexities impacting their capabilities (details are present in **Table 4**):

- MiT-B0: Smallest encoder with 32–256 channel counts, 4–2 patch resolution, 2 layers per stage, 1 head per layer, and fixed 8x MLP expansion ratios. Trades off feature learning and global context modeling for efficiency.
- MiT-B3: Greater capacity with 64–512 channel counts, 4–2 patch resolution, 3–18 layers per stage, 1–2 heads per layer, and flexible 8x-4x MLP expansion ratios. Balances efficiency and performance.
- MiT-B5: Prioritizes representational power over efficiency, having 3–40 layers per stage, 1–8 heads per layer, and larger width and depth for maximized global context modeling and rich feature learning.

2. Follow SegFormer decoder’s four main steps:

- Obtain feature maps from the four encoder stages and pass them through an MLP layer to modify channel dimensions (256, 768, and 768 for MiT-B0, MiT-B3, and MiT-B5).
- Up-sample or rescale features to a quarter of their original size and concatenate to build a feature map with 256 or 768 channels.
- Combine consecutive features using an MLP layer.
- Generate semantic segmentation predictions using another MLP layer and the merged feature.

Notable is that different encoder architectures lead to varying balances between model size, feature learning, and inference latency, causing distinctions in segmentation proficiency and efficiency.

### 2.8 Evaluation metrics

There are several common evaluation metrics used to assess the performance of segmentation models. These metrics help measure the accuracy and quality of the segmentation results.

#### 2.8.1 Pixel accuracy (accuracy): calculation of pixel-wise category counts

• Let G represent the ground truth image with correct category labels, and P represent the predicted image with category labels. Additionally, let H and W denote the height and width of the labelled image, respectively. P_ij_ signifies the count of pixels where the true label is category i, and they were predicted as category j. The calculation for P_ij_ is as follows, **Equation 1**:


(1) $$P_{ij}=\sum_{h=1}^{H}\sum_{w=1}^{W}\delta \left(G_{hw}=i\right).\delta \left(P_{hw}=j\right)$$

• where:

• ${\text{P}}_{\text{ij}}$ represents the count of pixels, where the actual category label is *i* in the ground truth image, and they are predicted as category *j* in the predicted image.

• $\sum_{\text{h}=1}^{\text{H}}:$ double summation. It iterates over the height (ℎ) of the label images.

• $\sum_{\text{w}=1}^{\text{W}}:$ another double summation, iterating over the width (w) of the label images. The width of the image is denoted by W.

* $\delta \left(G_{hw}=i\right)$ : this is the Kronecker delta function, which checks whether the pixel at coordinates (*h*,*w*) in the ground truth image (*G*) has the category label *i*. If the condition is true, $\delta \left(G_{hw}=i\right)$ equals 1; otherwise, it equals 0.

* $\delta \left(P_{hw}=j\right)$ : similarly, this Kronecker delta function checks whether the pixel at coordinates (*h*,*w*) in the predicted image (*P*) has the category label *j*. It equals 1 if the condition is true and 0 if it is false.

Pixels Accuracy calculates the fraction of correctly classified pixels in the entire image. It provides a measure of overall pixel-level accuracy. MPA and PA are expressed mathematically as follows, **Equation 2**:


(2) $$PA_{i}=\frac{P_{ii}}{\sum_{j=0\;}^{k}P_{ij}}$$

where:

$PA_{i}$ represents the Pixel Accuracy for category i .

$P_{ii}$ is the count of pixels where both the actual category label and the predicted label are i . In other words, it is the count of true positives for category i . These are the pixels that were correctly predicted as category i .

$\sum_{j=0}^{k}P_{ij}$ is a summation over *j* from 0 to *k*, where *k* represents the total category numbers (including background categories). It calculates the total count of pixels that are supposed to be category *i* in the ground truth image, regardless of whether they were predicted correctly or not.

#### 2.8.2 Mean pixel accuracy

Mean Pixel Accuracy, sometimes called Mean Accuracy, calculates the average accuracy of each class. It takes into account the class-wise accuracy and computes the mean, **Equation 3**.


(3) $$MPA=\;\frac{1}{k+1}\sum_{i=0}^{k}PA_{i}$$

where:

$PA_{i}$ denotes the pixel accuracy of the i-th class, k refers to the total number of classes, and the “+1” accounts for the background class. Essentially, MPA averages the individual class accuracies, providing a holistic measure of segmentation performance considering all classes present in the dataset.

#### 2.8.3 Mean Intersection over Union (Jaccard Index):

Mean IoU, or Mean Intersection over Union, quantifies the extent of overlap between the predicted segmentation masks and the corresponding ground truth masks. In other words, it represents the ratio of the intersection for class *i* to the union for class *i*, **Equations 4**–**6**. Mean IoU is a valuable metric for evaluating the accuracy and precision of semantic image segmentation models (**Equation 7**), where the intersection for class *i* is, **Equation 4**:


(4) $$Intersection_{i}=\sum_{pixel}\left(P_{i}\cap^{\;}G_{i}\right)$$

Union for class *i* is given by, **Equation 5**:


(5) $$Union_{i}=\sum_{pixel}\left(P_{i}\cup^{\;}G_{i}\right)$$

$P_{i}$ and $G_{i}$ are the predicted and ground truth masks for class *i*, respectively.

The mathematical expressions for IoU is **Equation 6**


(6) $$IoU_{i}=\frac{p_{ii}}{\sum_{j=0}^{k}P_{ij}+\sum_{j=0}^{k}P_{ji}-p_{ii}}$$

where $\sum_{j=0}^{k}P_{ij}$ : This part sums up all the pixels that should be category *i* in the ground truth image, whether they were predicted correctly or not. It includes true positives and false negatives for category *i*. $\sum_{j=0}^{k}P_{ji}$ : Similarly, this part represents the sum of the count of pixels that were predicted as category *j* and are supposed to be category *i* in the ground truth image. It includes true positives and false positives for category *i.*

By subtracting $p_{ii}$ from $\sum_{j=0}^{k}P_{ij}+\sum_{j=0}^{k}P_{ji}$ removes the overlap between the true positives (common pixels between predicted and ground truth). This adjustment ensures that the IoU measures the proportion of the correctly predicted pixels relative to the total area that should be category *i* in the ground truth, excluding the overlap.


(7) $$mIoU=\frac{1}{k+1}\sum_{i=0}^{k}IoU_{i}\;$$

In the context of the segmentation task, k denotes the highest valid class label, while k+1 correspond to the overall sum of classes.

#### 2.8.4 FLOPs

Floating-Point Operations per Second (FLOPS) involves determining the number of floating-point operations a computer or a processor can perform in one second. FLOPS is a commonly used metric to measure the computational performance of hardware, such as CPUs, GPUs, or accelerators. GFLOPs (Giga-Floating-Point Operations per Second) represent one billion floating-point operations per second.

## 3 Results and discussion

The training and testing setup in this study involved specific hardware and software configurations. The computer used for this research is equipped with 10th generation Intel (R) Core (TM) i7–10870H processor, featuring 16 threads, 8 cores, a base clock speed of 2.21GHz, and a turbo speed of 5GHz. It is equipped with 16MB cache memory and supports a maximum memory size of 128GB (DDR4–2933). The graphics processing unit employed is the NVIDIA GeForce RTX3060, boasting 3840 CUDA cores and 6 GB of video memory. The operating system utilized is Windows 10, and the software stack includes PyTorch Lightning version 1.9.5, Python version 3.8, and CUDA version 11. PyTorch Lightning serves as a lightweight wrapper for PyTorch, streamlining the process of training and evaluating PyTorch models.

### 3.1 Segmentation visualization for various scenarios

To assess the efficacy of SegFormer, a comprehensive evaluation of the model’s performance was conducted on the testing set. The testing set was systematically divided into distinct subsets, each catering to different disease perspectives. These divisions were primarily based on the nature of the disease, the clarity of disease manifestations, and the density of disease regions.

**Figure 3:**
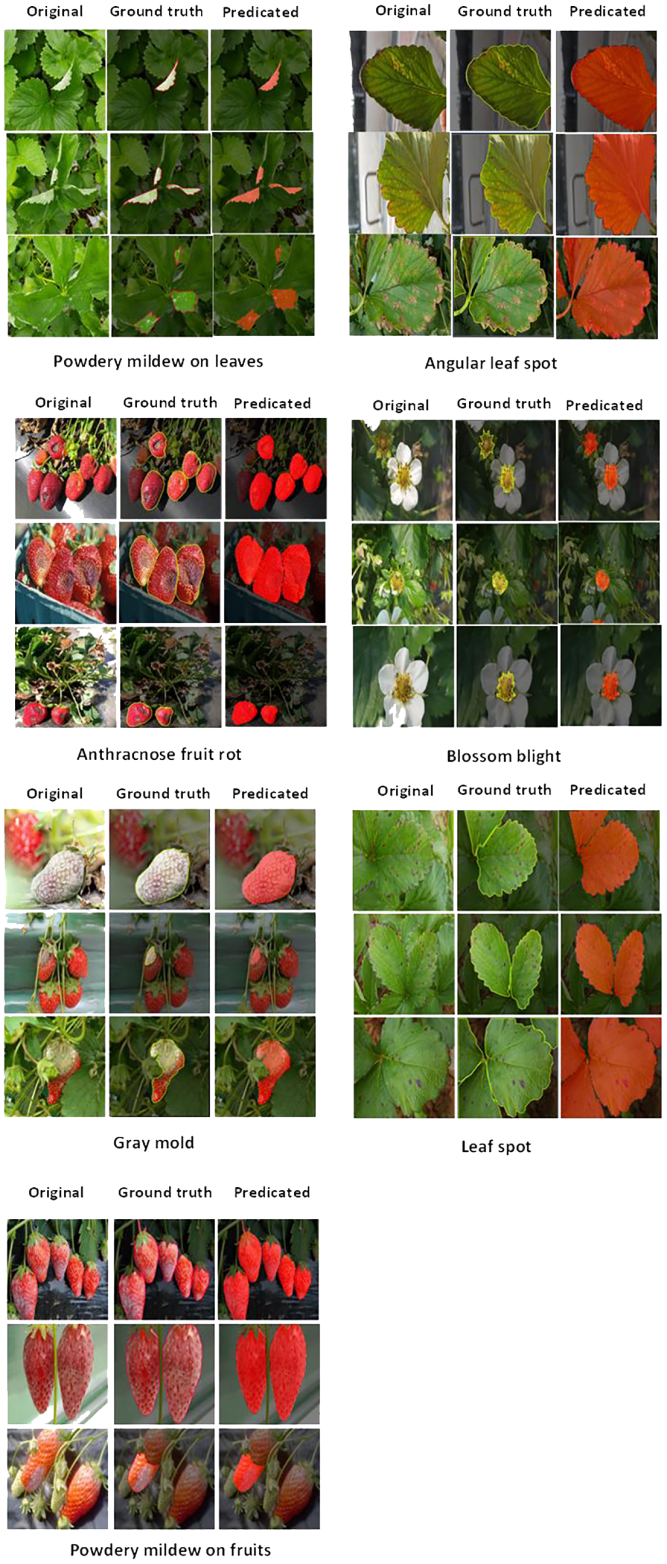
Visual representation of segmentation across various instances of strawberry diseases.

To evaluate the model’s ability to handle various disease types, three different disease perspectives for each disease were selected as research objects. The results of semantic segmentation of these diverse disease types are pictured at **Figure 3**. Moreover, for assessing the model’s performance in distinguishing between clear and blurry disease manifestations, samples representing both scenarios were selected, and their segmentation results were visually represented. Additionally, the study investigated the model’s competence in handling the sparseness and density of disease manifestations. Two samples were chosen to represent each scenario, and the segmentation results are visually presented in **Figure 3**. The visualized results demonstrate SegFormer’s remarkable ability to accurately identify and segment various disease types, consistent with manually labeled and segmented data, validating its effectiveness in semantic segmentation. **Table 5** presents a comparative analysis of various Mix Transformer encoder models in diagnosing six prevalent strawberry diseases. The tested models include MiT-B0, MiT-B3, and MiT-B5, evaluated on angular leaf spot, anthracnose fruit rot, blossom blight, gray mold, leaf spot, powdery mildew on fruit, and powdery mildew on leaves. Each entry contains the corresponding test loss, test mean pixel accuracy (MPA), test mean Intersection over Union (mIoU), computation complexity (GFLOPs), and the total estimated model parameter size in megabytes (MB). This detailed comparison helps assess each model’s performance, computational efficiency, and model size to guide developers and researchers towards an informed decision when selecting an appropriate model for specific strawberry disease detection tasks.

**Table 5:** Comparative performance analysis of various mix transformer encoder models.

Encoder type	Test-loss	Test-MPA	Test-mIoU	GFLOPs	Total estimated model params size (MB)
1. Angular leaf spot disease					
MiT-Bo	0.0669	0.95748	0.9238	0.846	14.859
2. Anthracnose fruit rot					
MiT-B0	0.1586	0.9321	0.8784	1.692	14.859
3. Blossom blight					
MiT-B0	0.0663	0.90891	0.8784	2.541	14.859
4. Gray mold											
MiT-B0	0.191	0.87751	0.7844	0.846	14.859
MiT-B3	0.095	0.9134	0.8567	26.76	188.896
MiT-B5	0.1299	0.895	0.835	37.41	338.380
5. Leaf spot					
MiT-B0	0.2023	0.9456	0.897	2.12	14.859
6. Powdery mildew fruit					
MiT-Bo	0.319	0.9296	0.8271	2.96	14.859
7. Powdery mildew leaf					
MiT-Bo	0.1726	0.691	0.6352	1.27	14.859
MiT-B3	0.1677	0.7753	0.6823	13.38	188.896
MiT-B5	0.1731	0.762	0.680	18.70	338.380
MiT-B3	0.0775	0.91293	0.8732	26.77	188.902

**Figure 4:**
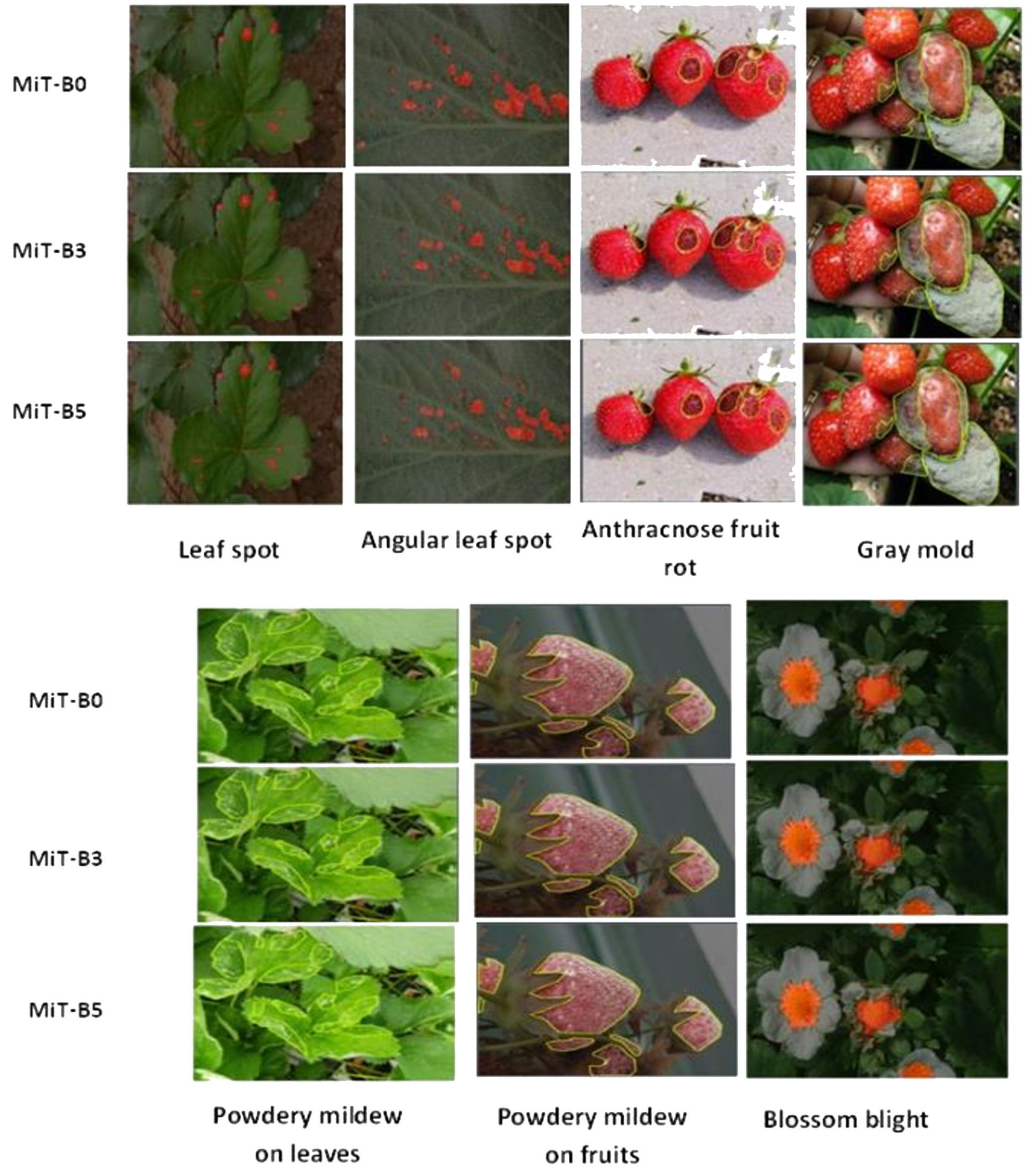
Visual representation of strawberry diseases segmentation process.

Based on the visual results for segmentation of various strawberry diseases **Figure 4** represents an explanation of the key observations:

- For Angular Leaf Spot disease, MiT-B3 and MiT-B5 perform well in identifying multiple spots on the leaves. MiT-B0 struggles with smaller spots. MiT-B5 delineates boundaries most cleanly.
- On Anthracnose Fruit Rot, all three encoders (MiT-B0, MiT-B3, MiT-B5) achieve accurate localization and segmentation of the disease regions. MiT-B5 produces the most precise segmentation boundaries.
- For Gray Mold, MiT-B3 and MiT-B5 accurately capture the diffuse disease regions, while MiT-B0 misses some portions. MiT-B3 provides finer segmentation.
- On Leaf Spot disease, MiT-B3 and MiT-B5 precisely identify and segment the multiple disease spots. MiT-B0 can detect some smaller spots. MiT-B5 offers the highest precision.
- For Powdery Mildew on Leaves, MiT-B5 clearly outperforms MiT-B0 and MiT-B3 in detecting the scattered powdery patterns. Its segmentation aligns closely with ground truth.
- On Powdery Mildew on Fruits, all encoders of MiT-B0, MiT-B3 and MiT-B5 achieve good localization. MiT-B5 provides the most accurate delineation.
- Finally, for Blossom Blight, all encoders effectively identify the affected flower regions.

As shown in **Figure 4**, MiT-B3 and MiT-B5 consistently outperform MiT-B0 across disease types, with MiT-B5 achieving the most precise segmentation in general. The results highlight the importance of selecting appropriate encoders matched to disease characteristics and use cases.

### 3.2 Boosting model training performance through augmentation techniques

**Figure 5:**
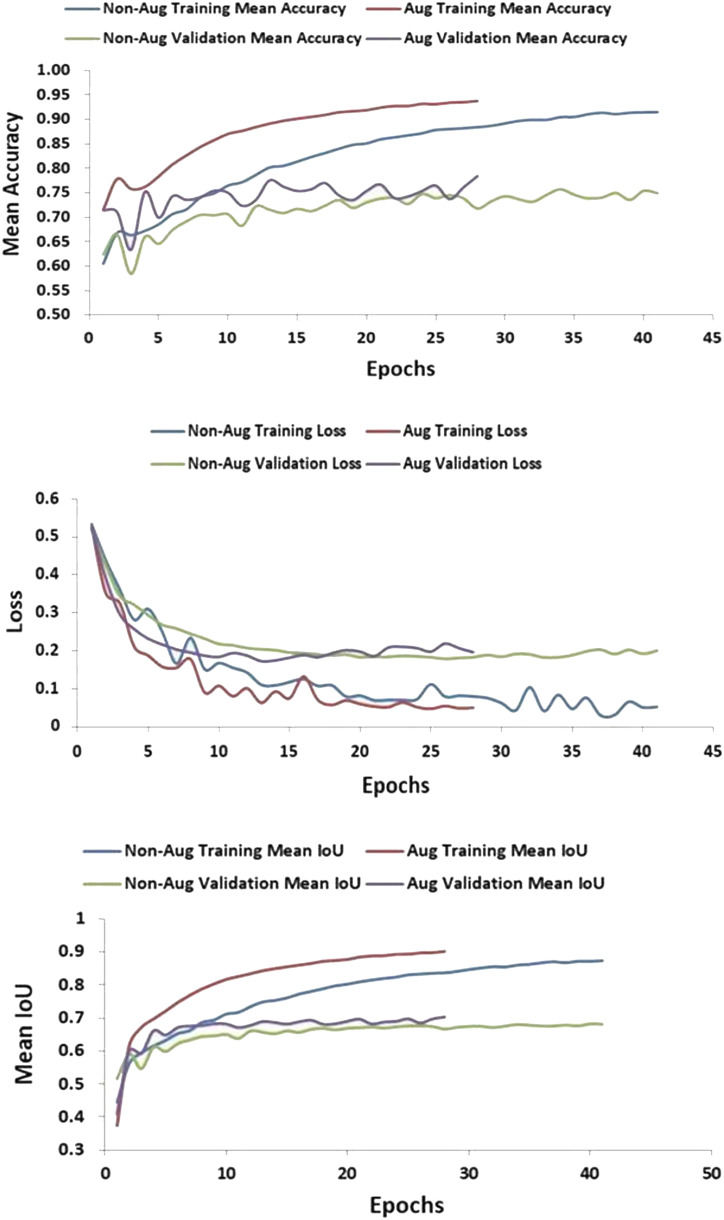
Optimizing Model Training Performance with Augmentation Methods.

The comparative results validate that data augmentation provided notable benefits for model training using the MiT-B3 encoder on the powdery mildew leaf disease dataset. Specifically, training with augmented data led to faster convergence evidenced by lower losses, reduced overfitting indicated by smaller gaps between training and validation metrics, more stable validation performance, and higher accuracy. For instance, by epoch 39 the training mean IoU reached 0.9 with augmentation versus 0.86 without. Meanwhile, the validation mean IoU improved gradually to 0.69 with augmentation compared to more fluctuation and ending at 0.68 without. Similarly, validation mean accuracy climbed to 0.76 with augmented data versus plateauing at 0.74 without. The consistent improvements in key metrics like loss, IoU, and accuracy demonstrate that introducing expanded diversity through augmentation techniques helped the model generalize better and boosted its capabilities, as shown in **Figure 5**.

The comparisons clearly validate that augmentation enabled superior training and segmentation performance, allowing the MiT-B3 encoder learn faster and achieve higher metrics on the powdery mildew leaf disease dataset.

### 3.3 Unleashing model potential: early stopping and checkpointing for precise strawberry disease detection

**Figure 6:**
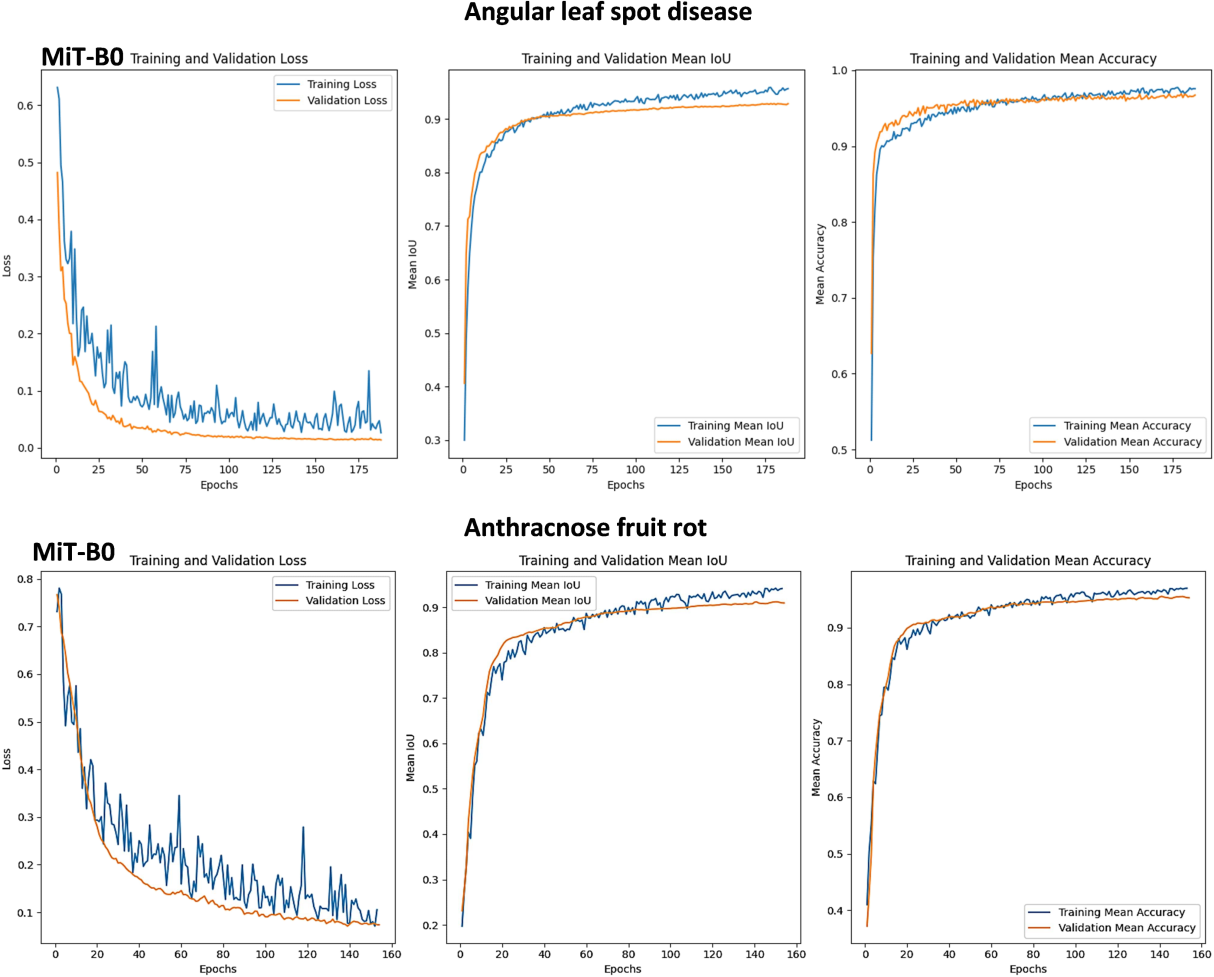
Graphs of Training and Validation Sets, along with Performance Metrics for SegFormer Evaluation on angular leaf spot and anthracnose fruit rot diseases using MiT-B0 Mix Transformer Encoders.

This section demonstrates the transformative power of early stopping and model checkpointing in optimizing a deep learning model for strawberry disease detection, as shown in **Figure 6**. By strategically employing these techniques, impressive results were achieved:

- Training and validation mIoU reaching 0.96 and 0.93, respectively, after 175 epochs.
- Remarkably low training and validation losses of 0.042 and 0.015 - a testament to the combined effectiveness of these methodologies.

**Early stopping**, a vigilant guardian, constantly monitored validation loss during training. When progress plateaued, it intervened, preventing overfitting and saving the model from memorizing training data instead of learning generalizable features.

**Model checkpointing** acted as a reliable safety net, preserving the best performing model versions throughout training. This invaluable technique ensured the progress due to potential training hiccups.

Together, these techniques fostered a harmonious balance between model complexity and generalization. The model effectively generalized to the unseen data, accurately identifying various strawberry diseases (Angular leaf spot, Anthracnose fruit rot, Blossom blight) under natural conditions.

The consistent performance across different diseases underscores the approach robustness. In synergy with innovative deep learning techniques, meticulous data preparation, and effective monitoring, early stopping and model checkpointing pave the way for real-world applications demanding high precision, like disease detection in agriculture.

### 3.4 Dissecting blossom blight detection: MiT-B3 outshines MiT-B0 in SegFormer models

**Figure 7:**
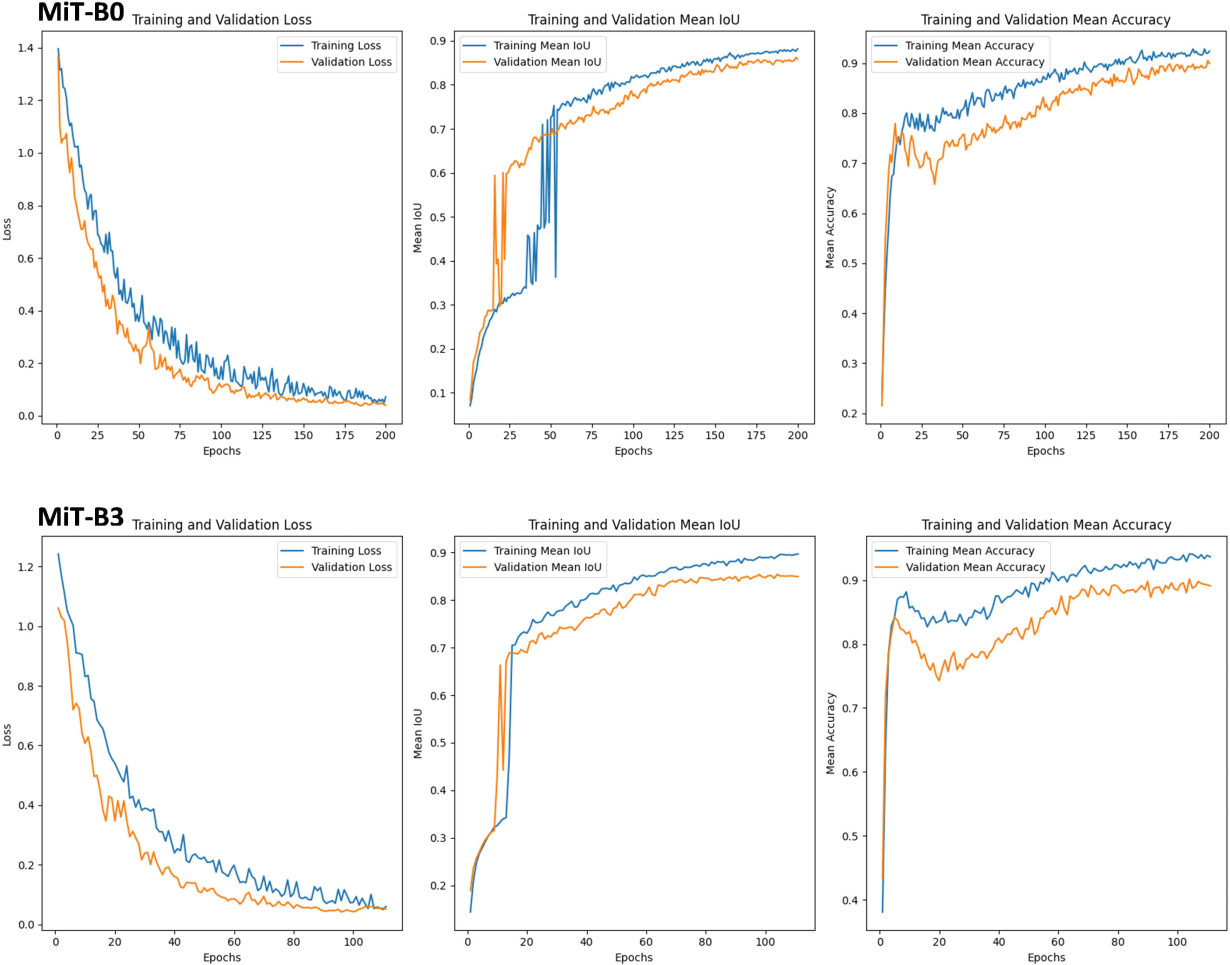
Graphs of Training and Validation Sets, along with Performance Metrics for SegFormer Evaluation on blossom blight disease using MiT-B0 and MiT-B3 Mix Transformer Encoders.

Understanding blossom blight in strawberries through deep learning is crucial for effective disease management. This section compares two prominent architectures, MiT-B0 and MiT-B3, within SegFormer models to see which encoder excels in detection, as shown in **Figure 7**. The results clarify key factors for choosing the right model for tackling specific diseases.

MiT-B0: While showing potential, consistency remains a hurdle. During training, its mean IoU (a measure of segmentation accuracy) fluctuates significantly. This suggests difficulty adapting to the disease’s diverse manifestations. However, the gradual rise in validation mean IoU indicates promising generalization to unseen data. Further investigation is needed to unlock MiT-B0’s full potential for consistent accuracy.

MiT-B3: This architecture outperforms in both rapid adaptation and stability. Training mean IoU experiences a remarkable jump from 0.34 to 0.7 within a single epoch, demonstrating efficient learning of disease features. Even after initial fluctuations, validation mean IoU stabilizes and steadily climbs, reaching 0.85. This signifies successful adaptation and consistent accuracy on unseen data, making MiT-B3 ideal for real-world scenarios. The choice of Mix Transformer encoder significantly impacts performance. While MiT-B0 shows potential, MiT-B3 dominates when it comes to swift adaptation and reliable detection. Its rapid learning and strong validation performance make it the clear winner for applications demanding fast adaptation and real-world disease detection.

### 3.5 Unveiling the gray mold buster: MiT-B3 reigns supreme in SegFormer models

**Figure 8:**
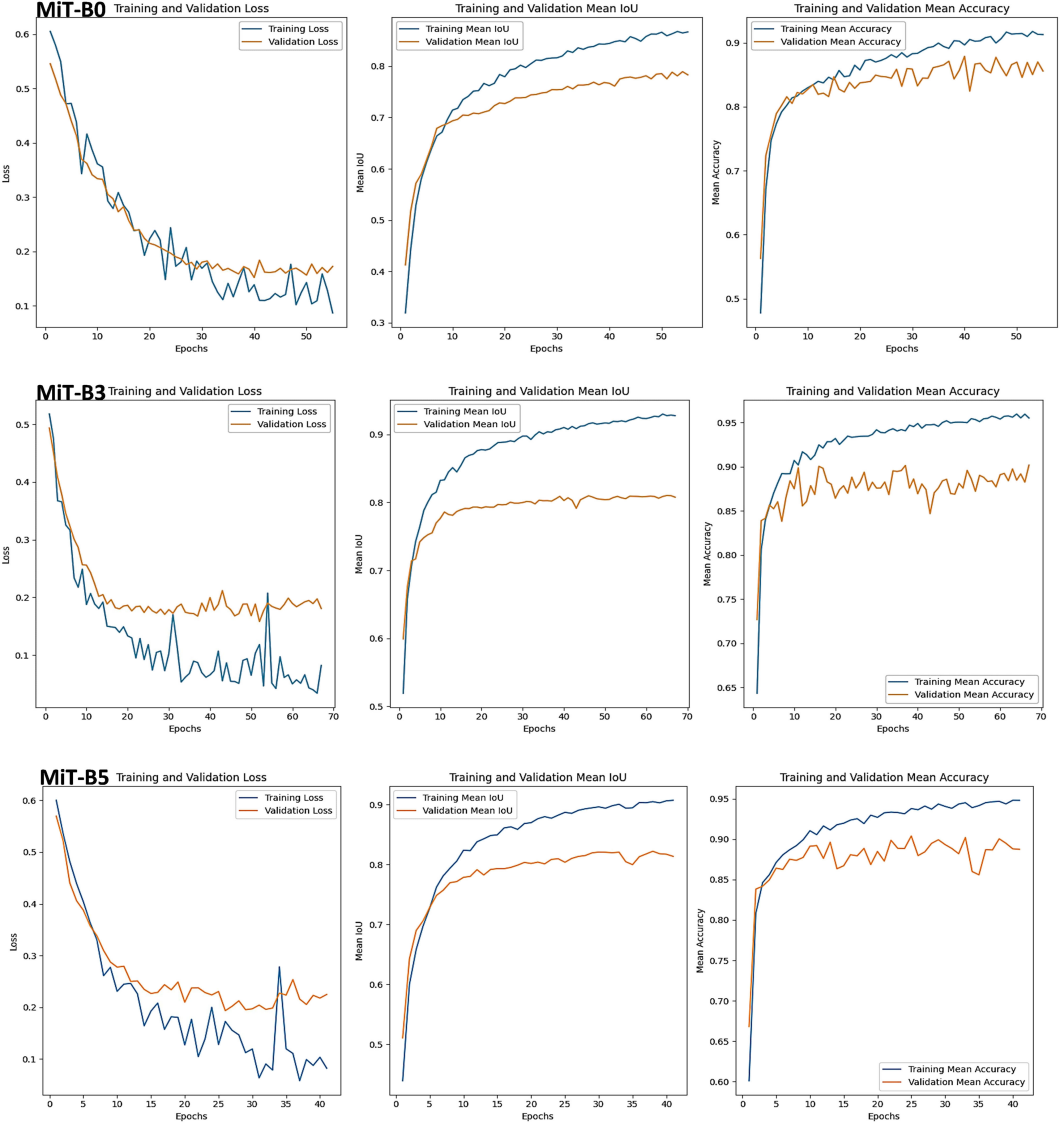
Training, Validation Sets, and Performance Metrics for SegFormer-Based Model Evaluation on Gray Mold Strawberry Disease using MiT-B0, MiT-B3, and MiT-B5 Mix Transformer Encoders.

Combating gray mold in strawberries requires effective detection tools. This section investigates three Mix Transformer encoders within SegFormer models - MiT-B0, MiT-B3, and MiT-B5 - to find the champion disease detective, as shown in **Figure 8**. The results hold valuable insights for both disease detection and model selection.

#### 3.5.1 MiT-B0

A solid contender, but with room for improvement. While converging well with similar training and validation losses (0.12 and 0.18), a lower validation mIoU (0.79) compared to training (0.87) implies possible overfitting. However, consistent accuracy across training and validation (0.91 vs. 0.87) shows promise.

#### 3.5.2 MiT-B3

Exceptional generalization and fitting are evident in its low training (0.045) and validation losses (0.19). High mIoU values for both training and validation (indicating ability to capture disease details) solidify its lead. Even on unseen test data, it scores a strong mIoU of 0.8567. Impressively high accuracy, especially on the test set, confirms its reliable gray mold identification under diverse conditions.

#### 3.5.3 MiT-B5

Training loss exhibits some instability, potentially impacting performance. While training mIoU is high (0.909), validation and test mIoU are slightly lower (0.82 and 0.835, respectively). This encoder demonstrates respectable scores, although lacks the consistency of MiT-B3. Its high training accuracy (0.95) is mirrored in validation and test sets (0.89 and 0.895), indicating potential but requiring further optimization.

#### 3.5.4 Key takeaways

• Encoder choice matters: MiT-B3 consistently outperforms the others in mIoU, accuracy, and convergence.

• MiT-B0 is well-balanced but susceptible to overfitting.

• MiT-B3 is the champion with exceptional performance and generalization.

• MiT-B5 shows potential, but requires refinement for stability.

The findings: For tackling gray mold, MiT-B3 proves to be the most effective encoder. Its exceptional performance and impressive generalization power make it an invaluable tool for accurate disease detection in real-world scenarios. This study underscores the importance of matching the encoder to the specific disease for optimal results, paving the way for improved strawberry protection and enhanced agricultural practices.

### 3.6 Detecting leaf spot and powdery mildew with SegFormer models

**Figure 9:**
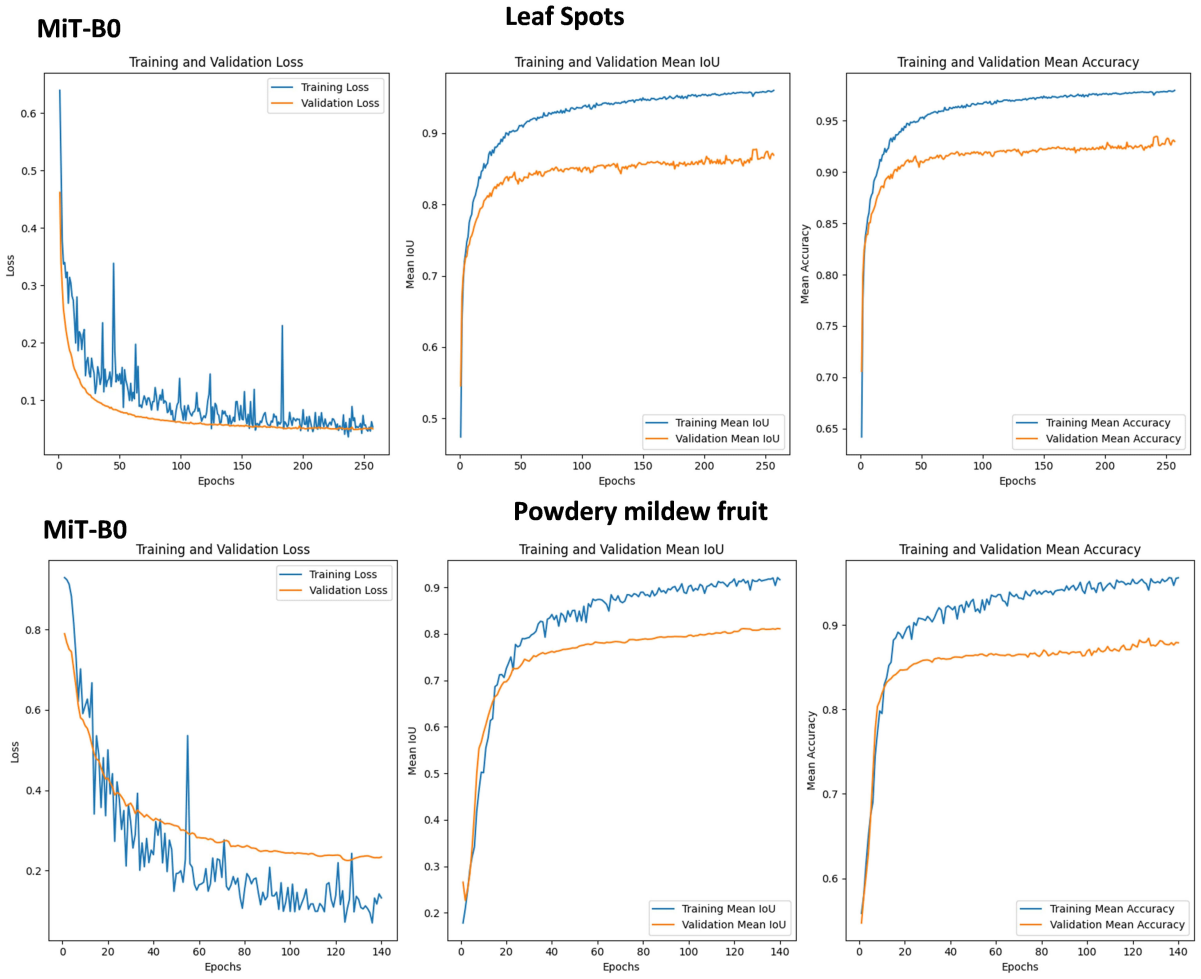
Graphs of Training and Validation Sets, along with Performance Metrics for SegFormer Evaluation on leaf spots and powdery mildew fruit diseases using MiT-B0 and MiT-B3 Mix Transformer Encoders.

This section explores the ability of MiT-B0, a Mix Transformer encoder, within SegFormer models to detect two distinct strawberry diseases: leaf spot and powdery mildew fruit disease (**Figure 9**).

#### 3.6.1 Leaf spot

• **Training loss:** Experienced two peaks, suggesting temporary difficulty due to disease complexity. However, it eventually reached a low value of 0.05.

• **Validation loss:** Steadily decreased and plateaued at 0.05, indicating consistent performance on unseen data.

• **Mean IoU:** Training mIoU reached a high of 0.98, while validation mIoU stabilized at 0.88, demonstrating effective learning and reliable detection.

• **Accuracy:** Both training and validation accuracy were high (0.98 and 0.93 respectively), confirming accurate disease identification.

**Powdery Mildew Fruit Disease:**

• **Training loss:** Fluctuated within 0.12 but peaked significantly at epoch 56. Ultimately, it decreased to 0.1.

• **Validation loss:** Showed a steadier decrease, plateauing at 0.22 and achieving a test loss of 0.319.

• **Mean IoU:** Training mIoU was high at 0.92, while validation mIoU was slightly lower at 0.81, indicating efficient learning but less accurate validation performance.

• **Accuracy:** Training and validation accuracy remained strong (0.98 and 0.88 respectively), with a test accuracy of 0.9296.

#### 3.6.2 Key takeaways

• **Adaptability:** The model successfully tackled both diseases, highlighting its potential for diverse applications.

• **Learning Power:** Consistent validation performance signifies effective learning despite training loss fluctuations.

• **Trade-offs:** Higher complexity (leaf spot) might cause temporary training challenges, but the model adapts and stabilizes.

MiT-B0 proves adaptable in detecting different strawberry diseases. While training loss may fluctuate with disease complexity, the model demonstrates its ability to learn, generalize, and achieve reliable detection, making it a promising tool for precision agriculture.

### 3.7 Decoding powdery mildew: finding the best AI detector with SegFormer models

**Figure 10:**
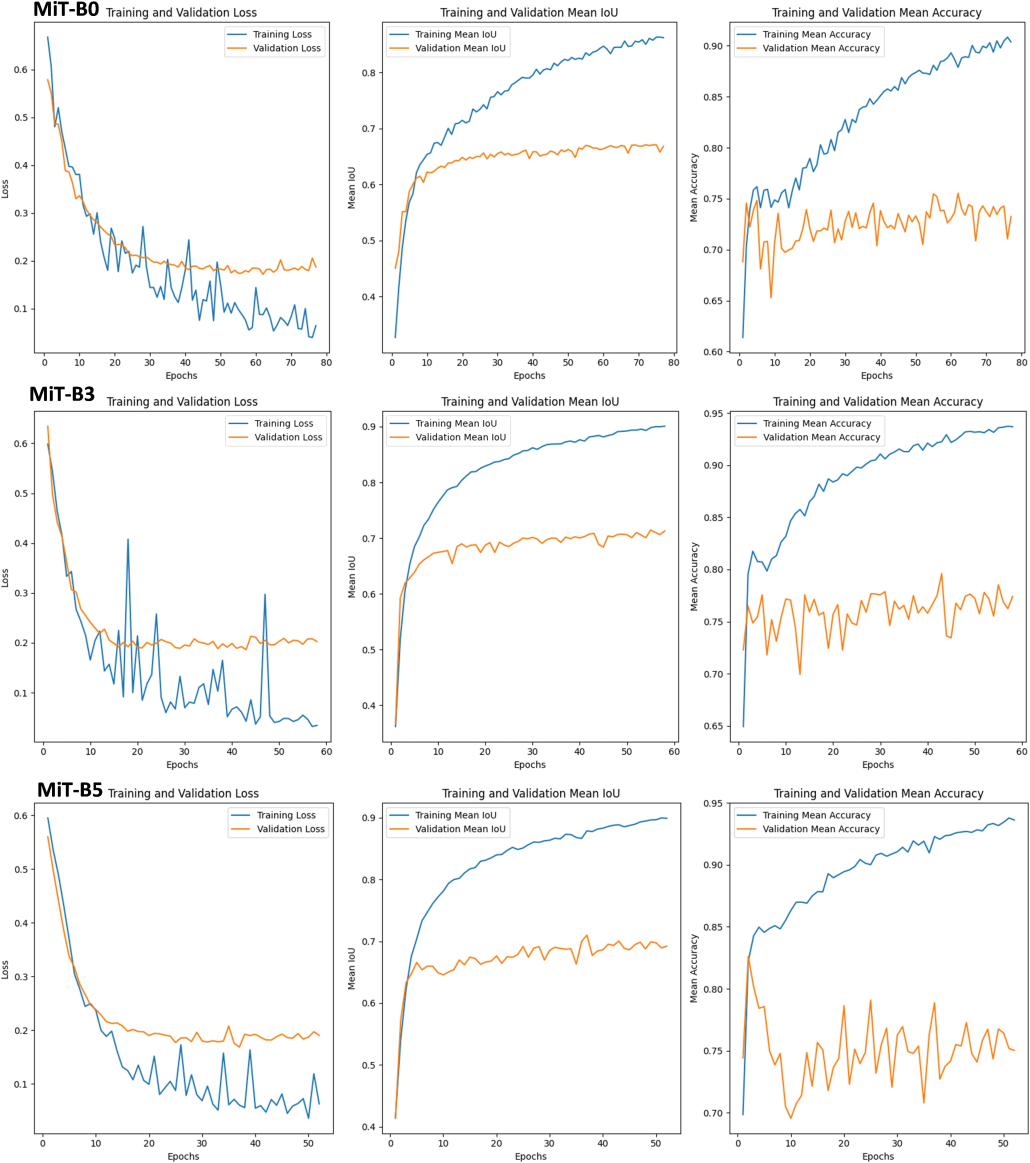
Training, Validation Sets, and Performance Metrics for SegFormer-Based Model Evaluation on powdery mildew leaf diseases using MiT-B0, MiT-B3 and MiT-B5 Mix Transformer Encoders.

This section delves into the performance of SegFormer models equipped with three Mix Transformer encoders (MiT-B0, MiT-B3, and MiT-B5) for detecting powdery mildew on leaves, as shown in **Figure 10**. Each model reveals unique behaviors and outcomes, offering valuable insights for choosing the right tool for the job.

#### 3.7.1 MiT-B0: efficient learner, room for growth

• Initial training demonstrates difficulties with loss fluctuations, revealing adaptation challenges.

• Validation loss stays stable, providing good generalization for unseen data.

• The encoder achieves a respectable mean IoU of 0.89 and accuracy of 0.92.

• Its low computational cost (1.269 GFLOPs) makes it a budget-friendly option.

#### 3.7.2 MiT-B3: speedy adapter, ideal for new disease variants

• It quickly adapts during training, boosting mean IoU to 0.9 and accuracy to 0.94.

• Validation performance also thrives, due to early stopping for efficient training in 60 epochs.

• It is ideal for scenarios demanding swift adaptation to novel disease variants.

#### 3.7.3 MiT-B5: fast learner, high accuracy (but pricey)

It converges rapidly with early stopping, reaching a high mean IoU of 0.9 and accuracy of 0.93 on both training and validation.

• It takes fewer epochs but demands more computational power (18.70 GFLOPs).

• It is perfect for situations where accuracy is paramount and resources are plentiful.

#### 3.7.4 Matching tool to task: a balancing act

• Encoder choice significantly impacts performance and adaptation speed.

• Complex diseases like powdery mildew benefit from MiT-B3’s quick adaptation.

• For efficiency-driven applications, MiT-B0 might be the best option.

Selecting the optimal Mix Transformer encoder specific disease, dataset, and resource constraints should be considered. Understanding the trade-off between computation, training time, and accuracy is crucial for real-world success. This detailed analysis empowers informed decision-making for disease detection tasks, ensuring the best AI tools application.

### 3.8 Comparative analysis with other segmentation models

To assess the effectiveness of the mix transformer encoders under study, several major segmentation models were trained and fine-tuned using the training and validation sets. **Table 6** below provides a comparative analysis of popular segmentation models and the proposed SegFormer variants. The comparison covers essential metrics like Total Parameters (M), mean Intersection over Union (mIoU), Mean Pixel Accuracy (MPA), and Flops (G). This comprehensive evaluation assists researchers and practitioners in determining the optimal model for their specific computer vision tasks, considering the trade-offs between model complexity, computational cost, and segmentation performance. Presented here are widely used models such as U-Net, DeepLabV3+, SegNet, and SETR, together with the newly proposed SegFormer configurations equipped with MiT-B0, MiT-B3, and MiT-B5 encoders.

**Table 6:** Comparative analysis with other segmentation models.

Model	Encode/Backbone	Total Params (M)	mIoU(%)	MPA (%)	FLOPs(G)
U-Net Ronneberger et al., 2015	MobileNetV2	24.33	63.9	69.3	45.23
	Vgg16	24.89	37.50	46.04	451.77
DeepLabV3+ Chen et al., 2018	DensNet-121	51.32	66.2	73.8	182.36
	Xception	54.71	32.10	51.25	166.87
SegNet Badrinarayanan et al., 2017	VGG16	29.46	57.0	62.7	284.10
SETR Zheng et al., 2021	ViT-Large	318.3	69.5	75.3	720.68
HRNet Wang et al., 2020	NA	29.54	31.02	53.75	79.96
ECA-SegFormer Yang et al., 2023	NA	4.04	38.03	60.86	10.64
PSPNet Zhao et al., 2017	MobileNetV2	15.4	61.1	68.2	84.9
	Resnet50	46.71	28.51	42.03	118.43
Proposed SegFormer	MiT-B0	**3.7**	**65.33**	**71.54**	**1.27**
	MiT-B3	**47.2**	**65.31**	**75.73**	**13.38**
	MiT-B5	**84.7**	**67.78**	**76.89**	**18.70**

The bold values highlight the model that performed best or had the maximum value for each evaluation metric. NA, Not Available.

As shown in **Table 6**, starting with model complexity - SegFormer demonstrates highly competitive performance with significantly lower model parameters compared to such state-of-the-art models like SETR and DeepLabV3+. For instance, even the largest MiT-B5 variant of SegFormer has 85% lesser parameters than SETR. This indicates SegFormer can match or exceed the capabilities of much larger models with far fewer parameters.

In terms of accuracy, measured by mean IoU and mean pixel accuracy, SegFormer consistently achieves outstanding results, outperforming classic models like U-Net, SegNet, and PSPNet. The MiT-B5 variant in particular exceeds DeepLabV3+ and comes close to SETR, which is remarkable given SETR’s massive size. This shows the representation power and generalization ability of SegFormer.

Finally, regarding efficiency, SegFormer requires significantly lower Floating Point Operations (FLOPs) compared to prior models like SETR and PSPNet. The smallest MiT-B0 SegFormer operates at less than 2 GFLOPs, enabling real-time inference on edge devices. Even MiT-B5 operates at nearly 4x lower FLOPs than SETR.

SegFormer establishes a new state-of-the-art in semantic segmentation across all key aspects - lower model complexity, greater accuracy, and higher efficiency. For strawberry disease segmentation, SegFormer provides the right balance of performance, accuracy, and efficiency as evidenced by the comparative analysis. This makes it the ideal choice to deploy in real-world agriculture applications.

## 4 Limitations and challenges

Despite the promising results and contributions of this research, there are certain limitations that require consideration. Addressing these constraints could give prospects for future exploration and improvements in the field of strawberry disease detection.

• **Limited scope of dataset:** Although the current study uses an adequately sized and diversified dataset, incorporating additional sources and increasing the volume of data could lead to more robust and generalizable models. Exploring multisource data fusion, combining images taken under different lighting conditions, geographical locations, and camera angles could further strengthen the model’s performance.

• **Impact of weather conditions:** Environmental factors, such as temperature, humidity, and sunlight exposure play a significant role in the appearance of strawberry diseases. Investigating the influence of these variables on model performance and accounting for dynamic weather conditions could result in more accurate and adaptable models.

• **Integration with Internet of Things (IoT) platforms:** Connecting the strawberry disease detection system with IoT devices, such as sensors and cameras installed in greenhouses, would facilitate real-time monitoring and decision-making. Further research could explore integrating the proposed model with IoT infrastructure for seamless implementation in agricultural settings.

• **Human-computer interaction for user feedback:** Developing intuitive user interfaces that allow users to provide feedback on model outputs could create opportunities for continuous learning and model improvement. Iteratively updating the model based on expert user inputs could result in more accurate and trustworthy systems.

## 5 Conclusion

This study has demonstrated the successful application of the SegFormer segmentation model for precise semantic segmentation of strawberry diseases, striving to enhance disease detection accuracy under natural acquisition conditions. The analysis of three distinct Mix Transformer encoders—MiT-B0, MiT-B3, and MiT-B5—has revealed their unique behaviors and benefits, catering to varying needs in disease detection applications. Adopting the novel SAM integrated into the Roboflow annotation tool enabled efficient annotation and preparation of a strawberry disease dataset, while rigorous augmentation techniques ensures the dataset’s quality and diversity. Balanced partitioning of the dataset into training, validation, and test subsets guarantees fair evaluation and optimized model performance. Implementing PyTorch Lightning, a potent deep learning framework, resulted in a finely tuned semantic segmentation model displaying impressive training and validation mIoU scores of 0.96 and 0.93, respectively. Moreover, SegFormer emerged victorious in comparative tests against other renowned segmentation models, outshining classical competitors such as U-Net, SegNet, and PSPNet in mean IoU and mean pixel accuracy. Crucially, SegFormer demonstrated its prowess operating with significantly fewer parameters and lower FLOPs than cutting-edge alternatives like SETR and DeepLabV3+, cementing its status as a compelling solution for practical agriculture applications. These findings hold great promise for the future of disease detection systems, suggesting that carefully chosen encoders paired with advanced models can deliver substantial improvements in accuracy, efficiency, and adaptability. As a consequence, researchers now have access to actionable insights for selecting the most suitable encoder in disease detection applications, propelling the field forward for further investigation. Future work in this domain includes multi-modal input integration, transfer learning across crops, online learning systems, scalable solutions, custom hardware development, benchmarking and standardization initiatives, open research platforms, and codebase creations. Ultimately, the goal is to establish robust, accessible, and adaptable AI technologies that empower stakeholders in the agricultural sector to make informed decisions and implement timely actions for sustainable food production.

## Data availability statement

Publicly available datasets were analyzed in this study. This data can be found here: https://www.kaggle.com/datasets/usmanafzaal/strawberry-disease-detection-dataset.

## Author contributions

WE: Conceptualization, Data curation, Methodology, Writing – original draft, Formal analysis, Investigation, Project administration, Software, Supervision, Validation, Visualization, Writing – review & editing. JG: Writing – review & editing, Data curation, Investigation, Methodology, Software, Validation, Writing – original draft. MS: Conceptualization, Data curation, Investigation, Methodology, Software, Writing – original draft. SA: Conceptualization, Formal analysis, Project administration, Resources, Validation, Visualization, Writing – review & editing. FM: Conceptualization, Formal analysis, Project administration, Resources, Visualization, Writing – review & editing. TE-M: Writing – review & editing, Conceptualization, Formal analysis, Project administration, Validation, Visualization. DB: Funding acquisition, Writing – review & editing, Investigation, Methodology, Software. DM: Data curation, Methodology, Writing – review & editing. MM: Investigation, Methodology, Writing – review & editing. YP: Methodology, Software, Writing – review & editing. SE: Conceptualization, Formal analysis, Validation, Writing – original draft. AE: Conceptualization, Formal analysis, Investigation, Validation, Writing – original draft. TE-H: Validation, Formal analysis, Writing – review & editing.
